# Supplementary material for: Investigating the Molecular Impact of GGMSC on Redox and Metabolic Pathways in Pancreatic Cancer Cells
Source: Antioxidants (Basel). 2025 Sep 25;14(10):1163. doi: 10.3390/antiox14101163 (PMC12561508; doi:10.3390/antiox14101163)
Supplement: Supplementary file 1 [file antioxidants-14-01163-s001.zip › antioxidants-3883042-supplementary.pdf]

Supplementary Figure legend:

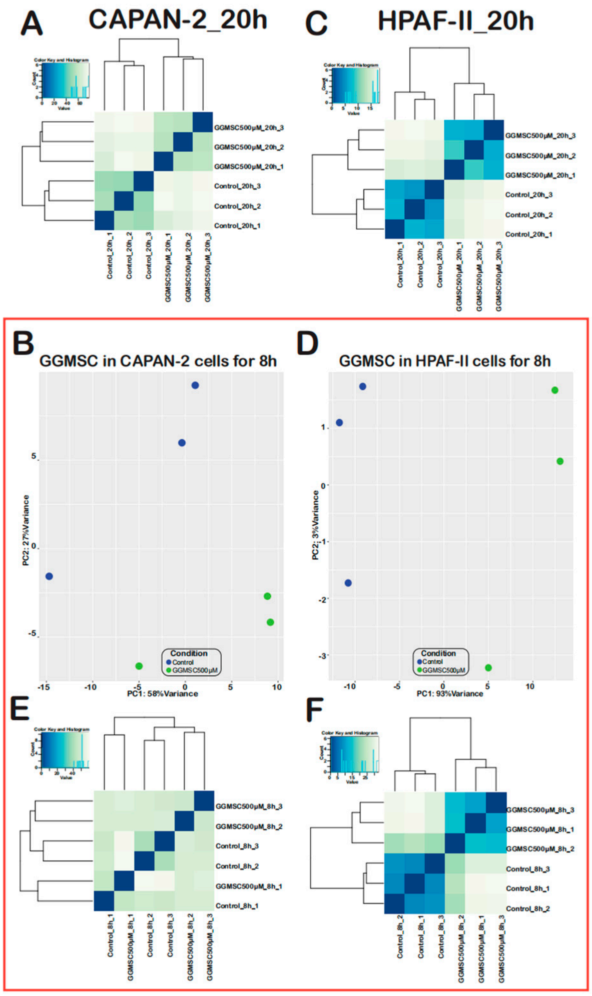

Supplementary Figure S1. Transcriptome-wide PCA and clustering of GGMSC-treated samples. (A–B) Hierarchical clustering heatmaps of CAPAN-2 **(A)** and HPAF-II **(B)** samples following 20 h GGMSC treatment (500 μM), based on Euclidean distances between sample expression profiles. **(C–D)** Principal component analysis (PCA) plots from Chipster analysis of global transcriptomes after 8 h GGMSC treatment in CAPAN-2 **(C)** and HPAF-II **(D).** Treated and control samples show partial separation, more pronounced in CAPAN-2. **(E–F)** Distance-based hierarchical clustering heatmaps of 8 h treated and control samples in CAPAN-2 **(E)** and HPAF-II **(F),** based on sample-to-sample expression distance. In all panels, clustering and PCA were performed using Chipster's default RNA-seq workflow. Color intensity reflects dissimilarity between samples.

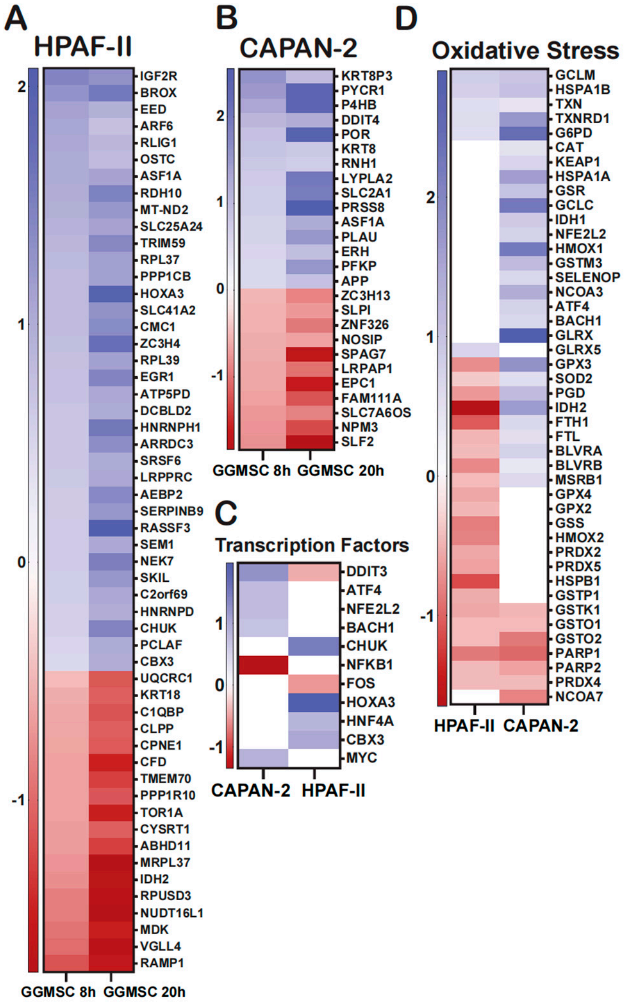

Supplementary Figure S2. Overlapping of transcriptional responses, differential regulation of transcription factors, and stress-related pathways by GGMSC. (A–B) Heatmap showing $\log_2$ fold changes of common DEGs in CAPAN-2 **(A)** and HPAF-II **(B)** cells following 500 μM GGMSC treatment for 8 h and 20 h. All displayed genes met the threshold of adjusted p-value < 0.01. **(C)** Heatmap of selected transcription factors showing stress-related activation in CAPAN-2 and survival-linked expression in HPAF-II. **(D)** Oxidative stress genes are selectively induced in CAPAN-2.

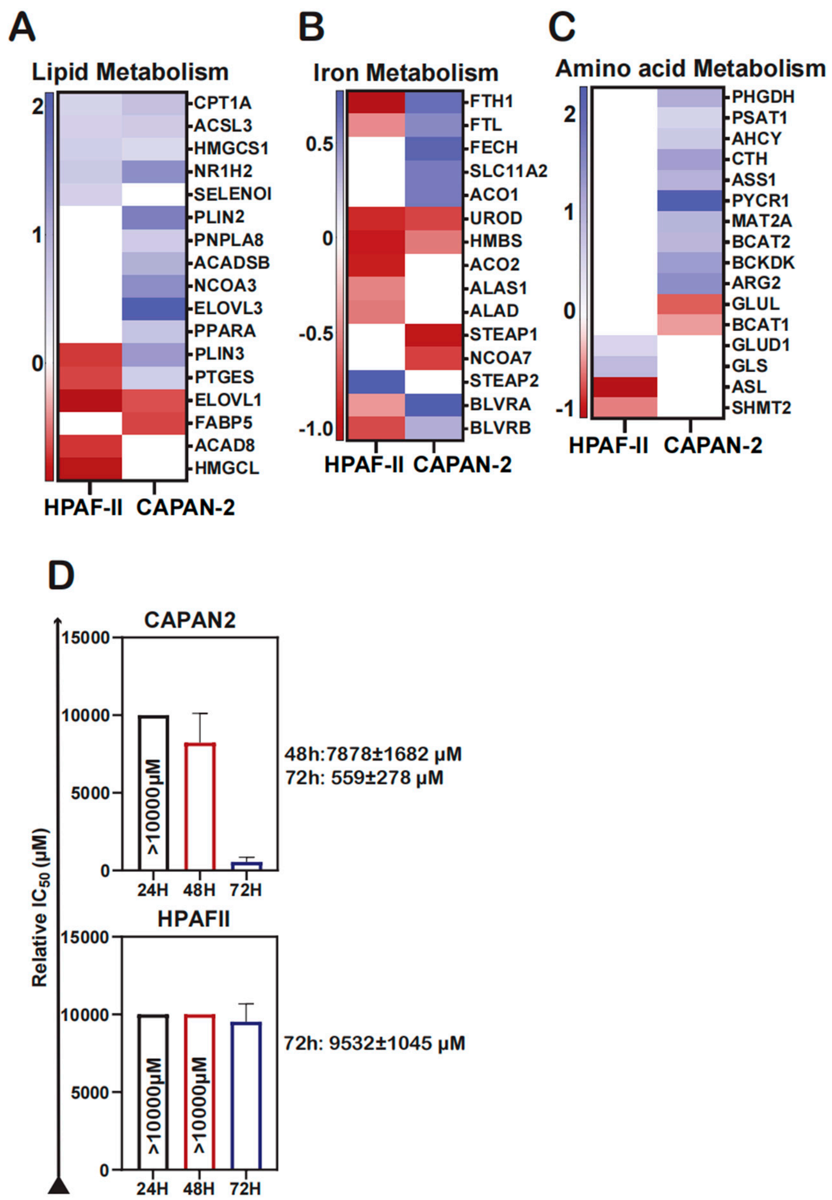

Supplementary Figure S3. Differential regulation of metabolic sub pathways by GGMSC. (A–C) Heatmaps of lipid metabolism **(A),** iron metabolism **(B),** and amino acid metabolism **(C)** genes show broader metabolic transcriptional activation in CAPAN-2. **(D)** Cytotoxicity assay demonstrating dose-dependent reduction in cell viability after GGMSC treatment; CAPAN-2 cells show greater sensitivity than HPAF-II, consistent with stress pathway activation.

Supplementary Tables

Supplementary Table S1. Oligonucleotides used.

Oligo	Sequence (5'-3')
**RT_VN30**	AAGCAGTGGTATCAACGCAGAGTACT$_{30}$VN
**TSO_II**	AAGCAGTGGTATCAACGCAGAGTACATrGrG+G
**PCR_Oligo_II**	AAGCAGTGGTATCAACGCAGAGT

Supplementary Table S2. PCR amplification conditions.

Step	Temperature	Time	Cycles
**Initial Denaturation**	98 °C	45 sec	1X
**Denaturation**	98 °C	10 sec	10X
**Annealing**	60 °C	10 sec	
**Extension**	72 °C	15 sec/kb	
**Final Extension**	72 °C	5 min	1X
**Hold**	4 °C	—	—

Supplementary Files:

Supplementary File S1: List of differentially expressed genes (DEGs) of CAPAN-2 and HPAF-II cells under all GGMSC treatment conditions.
